# Comparative Medicine

**Published:** 1906-06-23

**Authors:** 


### The Hospital

A Journal of

The Medical Sciences and Hospital Administration.

Vol. XL.—No. 1,031. SATURDAY, JUNE 23, 1906.

### Comparative Medicine.

To the layman who takes a sympathetic interest in the problems of medicine, and who has been accustomed at frequent intervals to experience a glow of satisfaction on reading of some new triumph over the forces of disease, it will probably come as a shock to be told on the authority of a Regius Professor of Physic that the promotion of health in the community is, even now, " left at a continual disadvantage." Such, however, is the concluding phrase of a letter which appeared in the *Times* of the 5th inst. above the signature of Professor Clifford Allbutt, and to the statement itself, as well as to the evidences advanced in its justification, medical men as well as laymen may reasonably be expected to lend an attentive ear. The conclusion is a serious one, and it is presented by an authority whose title and responsibility none will question. Further, the Professor's indictment—such, indeed, it is—not only involves a charge of inadequate appreciation of attempts to collect and co-ordinate knowledge bearing on the problems of disease, on the part of the public; it also proclaims the existence of a defect in the existing scheme of scientific research organisation of a nature so serious as to establish a general complexion of " muddle." And for this defect the medical profession must inevitably accept some measure of responsibility.

What, then, it will be asked, is this essential flaw which, in spite of half a century of brilliant progress, places numerous, and in some sense successful attempts at solution of problems of the highest national and scientific importance " at a continual disadvantage." It is, to put the matter briefly, the absence of arrangements necessary for a comprehensive survey of the whole field of disease and for the organised co-operation of the workers in the several departments of research. But to present this with due emphasis it is necessary to add that the problem of disease in man is not a solitary and detached question, to which physicians and pathologists, using these terms in their ordinary meaning, are the only possible and essential contributors. Even within this comparatively restricted area the claims of special lines of work are so imperative that the absence of effective supervision and co-ordination causes much loss and often leads to ill-judged perspective. The real field of survey, however, is much wider than the limits of human pathology." " Man, beast, and herb," as Professor Allbutt puts it, " we are all the product of one life," and though the misfortunes of our own species make a more immediate appeal to human sympathy and desire to help, these misfortunes can neither be fully understood nor successfully avoided until the relationships existing between them and the similar disasters which attend other and lowlier forms of life are completely explored. These disasters are, in truth, terrible and numerous enough. " Nature red in tooth and claw " is a tragic history, yet it cannot compete in horror with the continual devastation for which diseases are responsible—and diseases, be it remembered, which not only exact a severe economic penalty but probably conceal the secrets necessary to solve more than one of the problems of general pathology. It is true that some knowledge of these diseases already exists. But it is, at best, partial and incomplete, and, above all, it is isolated in compartments which " if not watertight are closely confined," so that its bearing on larger questions is not appreciated.

To remedy existing defects Professor Allbutt urges the establishment of a " general staff " of workers in comparative medicine. It is not a new institution which is needed but a scheme to secure systematic co-ordination of the results of existing operations. Further, what is true of the several branches of scientific work concerned with the problems of health and disease is not less true of the various public health authorities, which are " as Ptolemaic and fragmentary as our pathology." Here, on the same principle, is urged the establishment of a " Ministry of Health," short of which, " man, cattle, fish, and the fruits of the earth must still shift along as they can." It is not only comparative medicine, but organised comparative medicine that is offered as the solution of the existing loss and " muddle." To those who have followed Professor Clifford Allbutt's earlier utterances, his diagnosis of the present position of scientific medicine and his scheme of treatment are not new. He has urged his views again and again and with all his well-known incisive vigour and literary charm. Still more, efforts have been made at Cambridge to carry some of his plans into action, and it is melancholy to learn that these, so far from being extended, must now be relaxed from lack of funds. To meet this difficulty is not within the competence of the medical profession, however great its goodwill. But it does rest with every practitioner, as opportunity offers, to educate the public in a recognition of the truth that medical practice, whether curative or prophylactic, depends for its success on scientific observations conducted over the widest possible field and organised and directed under efficient guidance.

